# Management of Suprachoroidal Hemorrhage during Phacoemulsification: A Comprehensive Review

**DOI:** 10.3390/medicina59030583

**Published:** 2023-03-15

**Authors:** Ana Flores Márquez, Facundo Urbinati, Carlos Rocha-de-Lossada, Juan Ángel Moreno Gutiérrez, Mihnea Munteanu, Mariantonia Ferrara, Joaquín Fernández

**Affiliations:** 1 Department of Ophthalmology, Hospital Costa del Sol, 29603 Marbella, Spain; 2 Department of Ophthalmology, Hospital Regional Universitario de Málaga, 29010 Malaga, Spain; 3 Faculty of Medicine, Universidad de Málaga, 29016 Malaga, Spain; 4 Qvision, Ophthalmology Department, VITHAS Almería Hospital, 04120 Almería, Spain; 5 Ophthalmology Department, VITHAS Málaga Hospital, 29016 Malaga, Spain; 6 Departamento de Cirugía, Universidad de Sevilla, Área de Oftalmología, Doctor Fedriani, 41004 Seville, Spain; 7 Department of Ophthalmology, Victor Babes University of Medicine and Pharmacy, 300041 Timișoara, Romania; 8 Manchester Royal Eye Hospital, Manchester M13 9WL, UK

**Keywords:** cataract surgery, phacoemulsification, suprachoroidal hemorrhage, expulsive hemorrhage, visual prognosis

## Abstract

Suprachoroidal hemorrhage (SCH) is a rare and sight-threatening complication of various intraocular surgeries, including cataract surgery. Although the rate of SCH complicating cataract surgery has decreased in the era of phacoemulsification, most likely due to smaller self-sealing incisions and modern equipment, it remains a challenging complication to manage. The aim of this review is to summarize the current evidence of the pathophysiology and management of SCH complicating phaco surgery. A literature review was performed using the PubMed database searching for diagnosis, therapy, and management of SCH during phacoemulsification. The evidence available on the optimal management of this condition is low, and there is no consensus so far. An early diagnosis is thought to be essential to avoid progression to the devastating stage of expulsion of intraocular contents (expulsive hemorrhage). Sudden intraoperative anterior chamber shallowing, red reflex loss, and a significant increase in intraocular pressure are highly suspicious for this severe complication. A fundus examination and ocular ultrasound are crucial to confirm the diagnosis and, if it is confirmed, stabilize the globe immediately. The initial therapeutic approach includes aggressive topical and systemic medication focused on controlling ocular inflammation and intraocular pressure, whereas the timing and the indications of surgical intervention remain controversial.

## 1. Introduction

Suprachoroidal hemorrhage (SCH)—namely, a hemorrhage involving the suprachoroidal space—is a rare but severe complication reported in association with several intraocular surgeries, including glaucoma filtration procedures, pars plana vitrectomy (PPV), keratoplasty, and cataract surgery [1,2,3,4]. It is thought to be related to acute changes in intraocular pressure (IOP), either during surgery (acute SCH) or postoperatively (delayed SCH), causing the rupture of the ciliary arteries or smaller vessels and the subsequent accumulation of blood in the suprachoroidal space [5,6]. 

Cataract surgery is the most frequently performed intraocular surgery in the world [7] and the surgical technique has dramatically improved over the last few decades, mainly due to optimized phacoemulsification fluidics as well as the development of microincisional surgery [2,7,8,9], resulting in a significant decrease in the rate of complications, including SCH [2,3,7]. Consistently, the estimated prevalence of SCH is around 0.13–4% during extracapsular cataract extraction (ECCE) and between 0.013 and 0.5% for phacoemulsification [8,9]. However, the optimal management of this condition, to try to prevent serious visual consequences, remains a matter of debate and can be challenging for surgeons, especially anterior segment surgeons [6,8].

Early signs of this devastating complication may go unnoticed; thus, one should be alert to the initial symptoms and perform an accurate diagnosis to prevent the progression of SCH. Moreover, different therapeutic approaches have been reported, but there is no agreement on which of them is the best to ensure a successful outcome [5]. 

In this light, we reviewed the current evidence regarding the pathophysiology and management of SCH during phacoemulsification. Based on this, we summarized the information in an algorithm for the intraoperative management of SCH. 

## 2. Materials and Methods

A literature review was carried out using the PubMed platform using the terms “Expulsive hemorrhage” AND “Suprachoroidal hemorrhage” in combination with keywords such as “Cataract surgery” and “Phacoemulsification”. Due to the low incidence of these complications and with the aim to conduct a comprehensive review, we included both prospective or retrospective studies, case series, and case reports published up to December 2022. Only articles written in English were included.

The search findings were as follows:

- “(Expulsive Hemorrhage) OR (Suprachoroidal Hemorrhage)” AND “(cataract surgery)”: 105 articles.
- “(Expulsive Hemorrhage) OR (Suprachoroidal Hemorrhage)” AND “(Phacoemulsification)”: 77 articles.

A review of all the identified abstracts published in English was assessed. A total of 31 unique abstracts were considered for the review and 125 articles were excluded because they were not relevant for the review. All articles were carefully read, and the respective references were cross-matched to identify 12 more articles that had not been included in the initial search, resulting in a final number of 43 articles.

The exclusion criteria were as follows: (1) Articles dealing with suprachoroidal hemorrhage without referring to cataract surgery. (2) Articles referring to cataract surgery not performed using the phacoemulsification technique. (3) Studies in which suprachoroidal effusion/suprachoroidal hemorrhage was not confirmed. Duplicated articles were also disregarded.

## 3. Pathophysiology

Two types of SCH can be distinguished if the event happens during or after surgery: acute SCH (ASCH) and delayed SCH (DSCH) [6]. In general, the creation of surgical wounds cause IOP fluctuations during or after surgery. In patients with a stable vascular condition, these changes are compensated without adverse events; in those with a compromised vascular status, these fluctuations may alter the suprachoroidal space and result in SCH [10]. Numerous factors have been proposed to affect the choroidal perfusion of the eye, resulting in uncompensated changes in IOP and increased hemorrhagic risk such as high blood pressure, arteriolar sclerosis, increased retro-orbital pressure, and the use of preoperative vasoactive medication [10,11,12].

**Figure 1:**
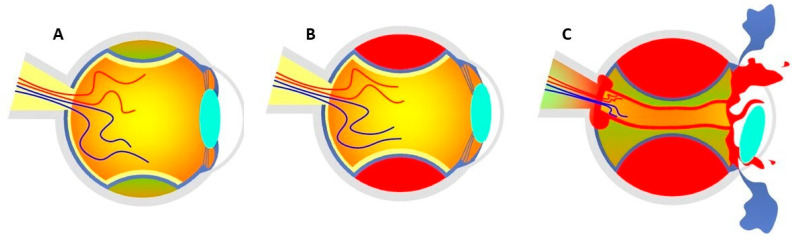
(**A**) Accumulation of blood serum in the choroidal layers at the stage of CE. (**B**) Shallow anterior chamber secondary to SCH without posterior pole involvement. (**C**) Expulsive hemorrhage that exceeds the equator, causing the expulsion of the entire intraocular content through surgical wounds. Figure inspired by the article of Savastano et al. [3].

The exact mechanisms leading to SCH are yet to be elucidated, but it has been speculated that IOP fluctuations, especially ocular hypotension, may produce an expanding choroidal effusion (CE) as the low IOP is not able to counteract the choroidal vessel pressure and leakage at this phase. If not controlled, CE may result in the overstretching and rupture of the ciliary arteries or choroidal vessels, with subsequent SCH. Ciliary nerves are also stretched, producing a severe radiating pain. In severe cases, the bleeding can exceed the equator and result in the expulsion of intraocular tissues through surgical wounds (expulsive hemorrhage (EH)) (Figure 1). However, the latter is supposed to be extremely rare using phacoemulsification [2,5,6,7,10]. Indeed, phacoemulsification with small corneal self-sealing incisions between 1 mm and 3 mm, a closed irrigation-aspiration (I/A) system, and faster postoperative wound healing minimize the risk of severe ocular hypotony and prevent the prolapse of intraocular contents [2,4,8,12,13,14,15]. For this reason, cataract surgery by phacoemulsification does not only decrease the risk of SCH, but prevents the extension of SCH to an EH if it occurs [2,8,14]. However, SCH during phacoemulsification is still a reported complication, and IOP oscillations during nucleus and cortex removal may particularly precede this event [8,9]. For instance, the presence of a non-self-sealing corneal wound during the removal of the phaco or I/A handpiece can cause a sudden IOP decrease [8,16]. In order to prevent the sudden depressurization of the eye at the time of the switch between the phaco and I/A handpiece, the use of an ophthalmic viscoelastic device to fill the anterior chamber before starting I/A is suggested [16]. Consequently, an increased incidence of SCH during or prior to I/A and intraocular lens (IOL) insertion has been reported [6,16]. Finally, secondary rapid depressurization of the eye occurring in failed coupling femtosecond laser-assisted phacoemulsification has also been suggested as an underlying mechanism in single cases [17].

### Risk Factors

Despite similar pathogenetic mechanisms, different risk factors have been identified for ASCH and DSCH complicating phacoemulsification, although the impact of a few of them is still controversial [11]. 

Regarding ASCH, a history of glaucoma, an elevated intraoperative pulse, and increased preoperatory IOP have been identified as independent risk factors [10,11,12]. The association between ASCH and cardiovascular medication as well as the factors causing fragility of the choroidal vasculature, including age and atherosclerosis, have also been reported to be significant [10,11,12,18]. Likewise, an increased retro-orbital venous pressure or regional anesthetic block may impede the flux of the vortex veins and, therefore, generate choroidal stasis and bleeding. Gentle orbital compression avoiding compressing devices following local anesthesia might soften the eye and, therefore, be protective against SCH [2,9,19,20]. Sub-Tenon’s block may have a low risk of SCH when compared with peribulbar or retrobulbar approaches [2,21]. Finally, situations with increased episcleral pressure—including carotid cavernous fistulas, vitreous prolapse, liver failure, the maturity of the cataract, or even a recent COVID-19 infection—have also been suggested as risk factors for this condition. Interestingly, anticoagulation has not yet been proven to be a risk factor for ASCH [9,12,21,22,23].

So far, the risk factors for DSCH associated with cataract surgery have not been specifically investigated. However, low postoperative IOP, systemic hypertension, and an older age have been reported in cases of DSCH related to phacoemulsification and might, therefore, play a role [8,18]. Studies, including glaucoma surgeries, have identified low postoperative IOP, aphakia, hypertension, and anticoagulation therapy as main risk factors for DSCH in glaucoma surgeries [18]. A longer axial length, the presence of rhegmatogenous retinal detachment, extensive intraoperative photocoagulation, and postoperative emesis have been shown to increase the incidence of DSCH in PPV [18]. Finally, high myopia and cardiovascular disease have been strongly correlated with limited SCH after intracapsular cataract extraction (ICCE) and glaucoma filtrating procedures, and have also been documented in cases of SCH after phacoemulsification [11,18].

## 4. Diagnosis

Sudden anterior chamber shallowing (ACS) during surgery (especially at the stage of nucleus removal, I/A, or IOL insertion), along with red reflex loss, IOP increase, and pain are highly suspicious for ASCH [8,12]. Other warning signs include prolapse of the iris that cannot be repositioned, rupture of the posterior capsule with vitreous loss, and the spontaneous dislocation of the intraocular lens into the anterior chamber [5,13,24]. The loss of the red reflex with progressing black shadows observed under the operating microscope is quite specific to ASCH and might be the only sign in vitrectomized eyes in which the aqueous substance contained within the vitreous cavity may escape through the zonule fibers, resulting in the absence of other typical signs of SCH development [24]. However, initially, SCH tends to be limited and peripheral, with no alterations to the red reflex [8]. 

In the case of DSCH, patients typically experience a sudden onset of severe ocular pain with an acute reduction in visual acuity a few days after surgery. The IOP can be low, normal, or elevated [5,18]. ACS, vitreous prolapse, and loss of the red reflex might be present [5]. 

**Figure 2:**
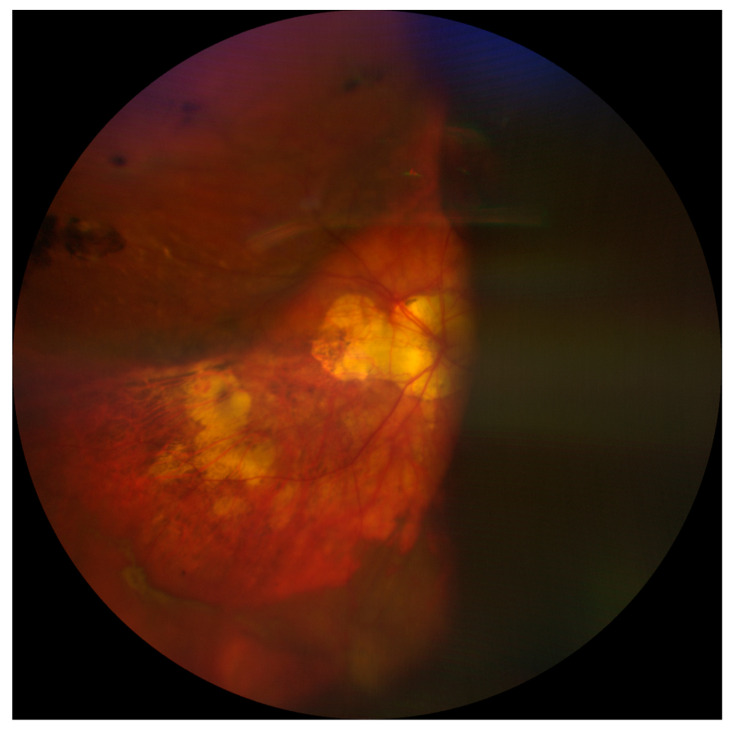
Choroidal detachment after cataract surgery in a highly myopic eye.

A fundus examination is far more sensitive than a red reflex assessment, and shows dome-shaped choroidal elevations affecting the equator or periphery, although these may extend posteriorly [4,8]. Hemorrhagic lesions do not transilluminate well, so the fundus appearance might also be useful in differentiating between CE and SCH [8]. Finally, B-scan ultrasonography plays a critical role in confirming the diagnosis and location of SCH as well as in assessing the state of the retina and vitreous gel. SCH appears as hyperechoic, solid-appearing choroidal detachments, with dense clotted hemorrhages and irregular shapes, which may decrease in density over time due to the liquefaction of the clots (Figure 2) [5]. For this reason, ultrasonography might also be helpful in monitoring and surgical planning because the liquefaction of the hemorrhagic clot has been suggested to improve the efficacy of drainage through sclerotomies [5,14]. In addition, performing B-scan ultrasonography can help to define the extent of SCH, in particular posterior pole involvement; this has been identified as a significant prognostic factor. Indeed, eyes with full-blown SCH (involvement of three or four quadrants) have been associated with an increased risk of a severe visual impairment (visual acuity less than 20/200) [16]. Finally, the presence of retrobulbar hemorrhage or an anesthesia-related globe perforation can be verified by B-scan ultrasonography [4,5].

### Differential Diagnosis

Capsular block syndrome (CBS) and fluid misdirection syndrome are both potential complications of cataract surgery that can mimic intraoperative SCH as both present with sudden ACS, pain, and the hardening of the eye; thus, they must be considered in a differential diagnosis [25,26].

In particular, intraoperative fluid misdirection syndrome can develop due to the inappropriate movement of a balanced salt solution posteriorly through the zonula fibers into or beside the vitreous gel and requires vitreous decompression [27]. Conversely, in the case of SCH, vitreous decompression can increase leakage and bleeding. Simply waiting for a few minutes may be helpful for the diagnosis as the anterior chamber (AC) usually deepens more rapidly in fluid misdirection syndrome than in SCH; a fundus examination provides the final diagnosis [6,25]. 

Intraoperative CBS occurs as a result of fluid sequestration in the capsular bag during hydrodissection. This happens due to the occlusion of the anterior capsule opening by the lens nucleus being displaced forward, resulting in posterior capsule stretching and IOP increase due to secondary angle narrowing. A gentle massage at this phase may reverse the situation whereas continuing the surgery could lead to posterior capsule rupture. A differential diagnosis between SCH and CBS can be based on the persistence of the red reflex examination and the typical occurrence after hydrodissection of the latter; if in doubt, fundoscopy can be performed [26]. 

Complications associated with regional anesthesia techniques—including retrobulbar hemorrhage and an inadvertent globe perforation—although rare, can occasionally be misdiagnosed as intraoperative or delayed SCH. Therefore, they must be considered in a differential diagnosis as well [4,5]. 

Retrobulbar hemorrhage is a rare but sight-threatening complication consisting of the accumulation of blood in the orbital space. Minor increases in volume may be compensated inside the orbit, whereas the rapid accumulation of blood in the retrobulbar space increases orbital pressure and results in the forward displacement of the eye (proptosis), pain, IOP increase, and potential ophthalmoplegia. Sudden pain and the hardening of the eye during or after surgery are common clinical findings of SCH, whereas proptosis, resistance to retropulsion, and a limitation of extraocular movement are considered to be differential signs between these two conditions. If in doubt, B-scan ultrasonography, a non-contrast computerized tomography (CT) scan, or magnetic resonance imaging (MRI) for an examination of the orbit may clarify the diagnosis [28].

An inadvertent globe perforation during retrobulbar or peribulbar block, especially in highly myopic patients, is a rare but potentially serious complication of ophthalmic surgery. Early signs include an IOP increase, if there is an inadvertent intravitreal injection of anesthetic agents, and red reflex abnormalities, which may mimic SCH findings. However, severe pain coupled with a sudden loss of vision during the injection and before surgery should prompt ophthalmologists to consider a globe penetration instead of SCH. Perforation signs such as retinal tears, sub-retinal hemorrhage, retinal or choroidal detachment, and retinal toxicity may be observed during a fundus examination; B-scan ultrasonography is useful in assessing the damaged quadrant and correlating it with the one used for the point of insertion of the needle [29].

Finally, localized SCH has been reported to cause a diagnostic dilemma by presenting as a choroidal mass following phacoemulsification [30]. Despite being considered to be a relatively rare differential diagnosis of a choroidal mass lesion, typical SCH dome-shaped choroidal elevations on fundoscopy closely mimic other life-threatening conditions such as choroidal melanoma and metastasis; thus, patients must be properly investigated. The key clinical findings of limited SCH include pain, overlying choroidal folds, and a spontaneous resolution; melanoma-like orange pigment is usually absent. Moreover, a meticulous analysis of multimodal imaging has been proven to be successful for differentiating between SCH and various choroidal mass lesions. Hyporeflective choroidal masses with a smooth anterior surface and an elevation of the retino-choroidal complex but no sub-retinal fluid on optical coherence tomography (OCT) as well as normal autofluorescence patterns are suggestive findings of SCH. Both fundus fluorescein angiography (FFA) and indocyanine green angiography (ICG) are useful for ruling out an intrinsic circulation; their features are usually unremarkable in SCH lesions, although late optic disc staining can be seen as a consequence of postoperative inflammation. Lastly, limited DSCH may present on B-scan ultrasonography as a mass with high surface reflectivity and low internal reflectivity. Invasive investigations and an extensive systemic workup should be considered in cases of non-resolution or when the diagnosis is uncertain [30].

## 5. Management

**Figure 3:**
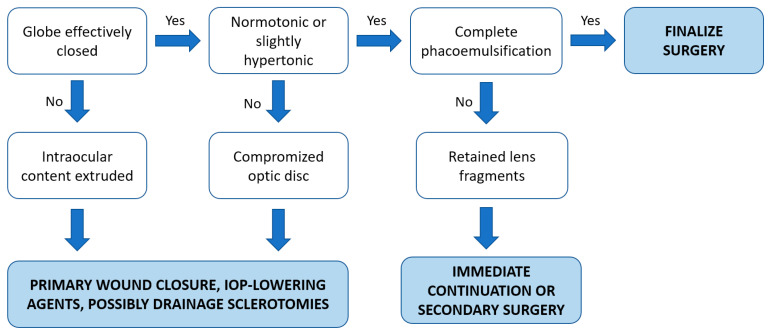
Proposed therapeutic algorithm of the intraoperative management of SCH.

Based on the current evidence, we summarized our approach for the intraoperative management of SCH into a simple algorithm, as mentioned in Figure 3.

### 5.1. Prophylactic Measures

As cataract surgery is widely accepted as an elective procedure, several authors have suggested that adopting certain preventive measures that may be performed before, during, or after surgery could avoid the occurrence of SCH [1,5,13]. 

First of all, patients recommended for cataract surgery, especially when considered to be high-risk cases, should be thoroughly evaluated in advance, paying particular attention to both systemic and ocular risk factors for SCH [1,5,13]. In this regard, a comprehensive examination should be undertaken, looking for cardiovascular disease, liver failure, coagulation abnormalities, and the use of cardiovascular medication [5]. Moreover, although not specifically addressed in the literature of phacoemulsification-related SCH, risk factors identified for glaucoma and vitrectomy-related SCH such as anticoagulant therapies should be taken into consideration as well. The bleeding risk should be assessed before the surgery through a complete blood count and coagulation panels; blood values with an emphasis on INR levels should be studied and stabilized before the procedure [1,13]. Finally, ocular risk factors, including high myopia and a history of glaucoma or IOP increase, should be documented and treated accordingly [1,5].

Perioperative measures for SCH prevention include the aggressive medical management of high IOP with intravenous hyperosmotic agents or carbonic anhydrase systemic inhibitors, if necessary; compressive devices should be avoided. An elevated intraoperative pulse and hypertension should be controlled, and the instillation of topical phenylephrine eye drops should be restricted to avoid systemic hypertension [5].

Postoperatively, the patient should be instructed to avoid Valsalva-type maneuvers as well as eye trauma or pressure. Postoperative inflammation and hypotonia should be assessed and vigorously treated [5].

### 5.2. Intraoperative Management

If acute SCH is suspected, immediate pressurization of the eye is required in order to raise the IOP to a level sufficient to stop the bleeding [5,14]. This is usually accomplished by either direct digital pressure or the suturing of surgical incisions. However, the value of suturing self-sealing incisions such as those typical of current phacoemulsification techniques has been questioned by a few authors [5,6,21]. Reformation of the AC, either by saline or air injection, may also be beneficial and prevent a vitreous prolapse [5]. Moreover, removing the eyelid speculum can lessen the pain and contribute to preventing the expulsion of ocular content by reducing direct pressure to the globe [5,6]. Moving the patient to either a reverse Trendelenburg (head being elevated) or semi-orthostatic position may also be useful to decrease the severity of the bleeding, potentially allowing the completion of the surgery as the vasculature of the cranial district lacks valves and the choroidal flux is redirected using this maneuver [6,13]. Osmotic agents (such as intravenous 20% mannitol) for reducing intraocular pressure, sedation for agitated patients, and medication for lowering systemic blood pressure are considered to be effective acute therapies for SCH, in particular if the surgery is resumed [5,6,13,20]. 

If the eye can be effectively closed and left normotonic or slightly hypertonic, further treatment is not necessary during surgery in most cases of SCH [5,13]. If possible, when suitable eye conditions are restored, resuming and finishing the surgery would be an ideal scenario in cases with incomplete phacoemulsification as it may have reduced the intraoperative risk [6,20]. Otherwise, especially if the intraocular contents cannot be reposited or a high IOP threatens the optic nerve, SCH drainage through posterior sclerotomies (PS) may be indicated [5,13,14]. Nonetheless, the long-term advantage of posterior sclerotomies performed intraoperatively remains controversial because the tamponade effect may be lost due to the acute drainage, potentially leading to the reappearance of bleeding [5]. In addition, a significant extension of the hemorrhage was described after the execution of immediate sclerotomies in an ex vivo rabbit model of non-expulsive massive SCH, raising concerns about the potential benefits of intraoperative sclerotomies and highlighting the primary importance of the immediate closure of the eye [31].

### 5.3. Postoperative Management

Regardless of the cause, the early management of SCH is usually similar and entails an aggressive medical therapy to control inflammation and intraocular pressure [5,13,14]. If the IOP is high, aqueous humor suppressants, both topically and orally, are required. Inflammation can be controlled by using topical steroids as well as systemic corticosteroids for severe cases. Pain can be managed with analgesics and adequate cycloplegia, although aspirin and non-steroidal anti-inflammatory drugs are contraindicated [5,13,14,32]. 

It has been reported that the majority of cases of phacoemulsification-related SCH tend to be limited; thus, medical management may be enough to achieve good visual outcomes [33,34]. Untreated SCH can lead to devastating consequences, including vision loss and phthisis bulbi [35]. Isolated cases of the spontaneous resolution of massive SCH with good visual outcomes have been reported; however, the prognosis of massive SCH is typically disappointing. In addition, massive SCH is frequently associated with retinal detachment and can lead to the deterioration of pre-existing ocular diseases [36]. Surgical management is usually considered in the following cases: kissing choroidal detachments; large choroidal hemorrhage that involves the macula; concomitant vitreous hemorrhage; vitreous incarceration; retinal incarceration or retinal detachment; inability to control concomitant elevated intraocular pressure with medical therapy; lens subluxation; and intractable severe ocular pain [5,34,35,36,37,38]. In particular, with regard to the SCH extent, it has been suggested that surgical intervention should be considered also in the absence of macular involvement if more than two quadrants are involved posterior to the equator [39]. The surgical approach to SCH entails transscleral or transconjunctival drainage procedures, potentially combined with PPV [5,13,21]. The presence of concomitant retinal detachment and/or vitreous hemorrhage favors the choice of a combined procedure [35]. The surgical procedure of drainage starts with a localized or 360 conjunctival peritomy, followed by the isolation of the recti muscles needed to expose the selected quadrants. Adequate intraocular pressure is maintained through an anterior chamber maintainer. Full-thickness drainage scleral incisions of about 2–3 mm length are created in a standard fashion 8–10 mm from the limbus in correspondence with the highest choroidal detachment, and mechanical pressure is exerted on the sides of the sclerotomies to promote the passive drainage of the suprachoroidal blood; in addition, during the drainage, the IOP can be increased in order to further facilitate it. The suture of the drainage sclerotomies may not be necessary [1,5,35]. 

A combination of SCH drainage with PPV is advisable in cases involving vitreoretinal traction, retinal detachment, vitreous incarceration, dense diffuse hemorrhage, or a dislocated cataract fragment [5,13,21]. Moreover, active aspiration techniques that utilize the aspiration strength of the vitrectomy suite and the infusion pump of the system have been proven to be successful in forcing hemorrhagic material out of the DS and could be useful in challenging cases [40]. Silicone oil is the preferred intraocular tamponade due to its long-term tamponade action and minimization of ocular hypotension, thus preventing potential rebleeding [21,35]. The intraoperative use of perfluorocarbon liquid has been suggested to stabilize the detached retina and displace the blood anteriorly, potentially easing the removal through anteriorly placed sclerotomies [1,5,21].

There is no consensus about the timing of the surgery so far [34]. Waiting preferably 10–14 days after the onset has been proposed to allow blood liquefaction to occur and, therefore, ease the drainage of the clots [5,13,34]. In this regard, serial ultrasounds can be indicated to monitor the hemorrhage and detect the liquefaction of the clotted suprachoroidal blood as well as to identify the site of the drainage sclerotomies based on the height of the hemorrhage [35]. It has been demonstrated in an experimental model of SCH in rabbit eyes that a choroidal detachment changes minimally in size during the first week whereas during the second week (7–14 days), there is the maximum liquefaction of the clotted suprachoroidal blood [31]. However, it also has to be considered that a longer duration of retinal apposition due to a kissing choroidal detachment has been associated with a poor visual outcome, and vitreous incarceration could lead to several complications, including retinal detachment [34,41]. In those cases that might benefit from an early approach, a tissue plasminogen activator (TPA) injection within the suprachoroidal space and a Sub-Tenon’s urokinase injection before PPV have been reported to be successful for assisting in the drainage of an organized clot prior to liquefaction, although the posology remains to be elucidated [41,42,43].

## 6. Limitations

Due to the low incidence of phacoemulsification-related SCH, the available literature is sparce and mostly includes studies with a low level of evidence such as case reports, small serial cases, or retrospective studies. It follows that the proposed summary algorithm for the management of SCH is not supported by strong evidence and is based on reportedly successful but not widely validated therapeutic approaches. To the best of our knowledge, randomized trials have not been published to date regarding this matter, likely also due to the complexity of setting-standardized inclusion criteria and the expected small sample size. 

## 7. Conclusions

SCH is a very rare and potentially severe complication of phacoemulsification. The current evidence suggests that early signs may be misdiagnosed or undetected and, therefore, a high index of suspicion is essential to detect and try to stop the progression of the bleeding. Sudden AC shallowing and an IOP increase even in the absence of red reflex abnormalities should prompt surgeons to consider SCH, pressurize the eye, and perform a fundus examination. B-scan ultrasonography is advisable in all cases, especially to properly differentiate between SCH and SCE, but is mandatory in cases of difficult fundus examinations. Intraoperatively, medical therapies and the positioning of the patient may restore suitable eye conditions to resume the surgery. Postoperatively, intensive medical care has been proven to be successful in most cases of limited SCH. If surgical management is indicated, the timing of sclerotomies and PPV remains to be elucidated. Further research is needed to standardize the management of SCH related to phacoemulsification, including the timing and the role of current available therapeutic approaches. 

## Data Availability

Not applicable.
